# Impact of treadmill running on distal femoral cartilage thickness: a cross-sectional study of professional athletes and healthy controls

**DOI:** 10.1186/s13102-024-00896-4

**Published:** 2024-05-06

**Authors:** Pouria Azami, Alireza Ashraf, Omid Yousefi, Alireza Hosseinpour, Aref Nasiri

**Affiliations:** 1 Department of Physical Medicine and Rehabilitation, Shiraz University of Medical Sciences, Shiraz, Iran; 2 Trauma Research Center, Shahid Rajaee (Emtiaz) Trauma Hospital, Shiraz University of Medical Sciences, Shiraz, Iran; 3 School of Medicine, Shiraz University of Medical Sciences, Shiraz, Iran

**Keywords:** Athletes, Cartilage, Exercise, Knee Joint, Running, Ultrasonography

## Abstract

**Purpose:**

This present study aimed to assess the impact of treadmill running on distal femoral cartilage thickness.

**Methods:**

Professional athletes aged 20 to 40 years with a history of treadmill running (minimum 75 min per week for the past three months or more) and age-, sex-, and body mass index (BMI)-matched healthy controls were recruited. Demographics and clinical features of participants were recorded. Athletes were divided into subgroup 1 with less than 12 months of treadmill running and subgroup 2 with 12 months or more of treadmill running. Distal femoral cartilage thicknesses were measured at the midpoints of the right medial condyle (RMC), right intercondylar area (RIA), right lateral condyle (RLC), left medial condyle (LMC), left intercondylar area (LIA), and left lateral condyle (LLC) via ultrasonography.

**Result:**

A total of 72 athletes (mean age: 29.6 ± 6.6 years) and 72 controls (mean age: 31.9 ± 6.7 years) were enrolled. Athletes had significantly thinner cartilages in the RLC (2.21 ± 0.38 vs. 2.39 ± 0.31 cm, *p* = 0.002), LLC (2.28 ± 0.37 vs. 2.46 ± 0.35 cm, *p* = 0.004), and LMC (2.28 ± 0.42 vs. 2.42 ± 0.36 cm, *p* = 0.039) compared with the control group. Furthermore, cartilage thickness was significantly thinner in subgroup 2 athletes compared with the control group in the RLC (2.13 ± 0.34 vs. 2.39 ± 0.31 cm, *p* = 0.001), LLC (2.22 ± 0.31 vs. 2.46 ± 0.35 cm, *p* = 0.005), and LMC (2.21 ± 0.46 vs. 2.42 ± 0.36 cm, *p* = 0.027); however, subgroup 1 athletes did not have such differences. There was a weak negative correlation between total months of treadmill running and cartilage thickness in the RLC (*r* = − 0.0236, *p* = 0.046) and LLC (*r* = − 0.0233, *p* = 0.049). No significant correlation was found between the distal femoral cartilage thickness at different sites and the patients’ demographic features, including age, BMI, speed and incline of treadmill running, and minutes of running per session and week (*p* > 0.05).

**Conclusion:**

Compared with healthy controls, professional athletes with a history of long-term high-intensity treadmill running had thinner femoral cartilages. The duration (months) of treadmill running was weakly negatively correlated with distal femoral cartilage thickness. Longitudinal studies with prolonged follow-ups are needed to clarify how treadmill running affects femoral cartilage thickness in athletes.

**Supplementary Information:**

The online version contains supplementary material available at 10.1186/s13102-024-00896-4.

**What Is Known?**

When physical activity is long-standing and vigorous, the risk of knee injury and subsequent initiation and progression of joint degeneration are higher. There are some differences in ground reaction force, kinetics, and kinematics between treadmill and non-treadmill running.

**What Is New?**

This unprecedented study found that athletes with a history of more than one year of high-intensity treadmill running had thinner cartilage thicknesses than the healthy controls in both knees.

## Introduction

Exercise and physical activity benefit the whole body, especially the musculoskeletal system. Different guidelines have encouraged people to undertake physical activity [1, 2], which is believed to enhance general health, decrease obesity, increase self-esteem, and increase longevity. A sedentary lifestyle and obesity can lead to osteoarthritis, while moderate exercise can prevent this condition. However, when it comes to vigorous physical activity performed by elite or professional athletes, hazards can be expected.

The increasing global popularity of motorized treadmills spans various settings, including sports clubs, gyms, medical facilities, and home users. About 14% of American runners and elite athletes utilize motorized treadmills as part of their training routine [3]. Treadmill running improves bone quality, although its effect on articular cartilage is questionable [4]. The overuse of joints can result in cartilage degradation [5, 6]. The risk of knee injury and subsequent incidents like the progression of joint degeneration may be elevated when physical activity is long-standing, vigorous, or a part of professional sport. In such conditions, the knee joint is exposed to higher impact or torsional loading levels. Thus, articular cartilage breakdown and loss, as the main events in the pathogenesis of osteoarthritis (OA), can be expected. A number of researchers have assessed the potential effects of physical activity on cartilage degeneration and subsequent OA. Murry et al. found that middle-aged men who had participated in excessive athletic activity during adolescence had a higher risk of hip OA development [7]. In contrast, Cymet et al. pointed out that long-distance running might protect the joint against degeneration [8]. Overall, all athletes of competitive sports are believed to be at a higher risk of incidence and progression of hip, knee, and ankle OA resulting in hospital admission [9].

In recent years, physiatrists’ use of musculoskeletal ultrasonography (US) has significantly increased. This modality is helpful in the diagnosis and treatment of musculoskeletal diseases and the evaluation of musculoskeletal structures. On the other hand, it is an inexpensive, non-invasive, convenient, and accessible modality compared with magnetic resonance imaging and computed tomography.

The impact of physical activity on cartilage condition has been extensively studied, yielding variable and occasionally conflicting conclusions. Research indicates that engaging in regular physical exercise such as running and cycling for as little as two weeks can result in reduced cartilage volume among young, healthy adults [10]. It has been shown that running is associated with decreased cartilage thickness in marathon runners compared with sedentary individuals. However, some investigations suggest that running may induce transient and reversible changes in knee cartilage morphology and composition [11–13]. Furthermore, evidence suggests that running does not contribute to the formation of new cartilage lesions and has minimal effects on foot and ankle cartilage [11, 12]. The specific type, intensity, and frequency of physical activity are key determinants in how they affect different articular cartilages [14]. Although the effect of vigorous physical activity on the cartilage condition has been evaluated previously, there is no study evaluating distal femoral cartilage thickness in professional athletes who undertake treadmill running by US. Therefore, this study was designed to assess the effect of high-intensity treadmill running on distal femoral cartilage thickness and to investigate the predisposing factors of cartilage thickness loss.

The long-term impact of sustained treadmill running on joint health remains uncertain. To address this gap in knowledge, we aimed to evaluate the distal femoral cartilage thickness among professional athletes engaged in treadmill running using US. Consequently, this study aims to investigate the effect of high-intensity treadmill running on distal femoral cartilage thickness and explore potential factors contributing to cartilage thickness loss.

## Methods

This study is reported following the STROBE guidelines [15].

### Study participants

Professional athletes aged 20–40 years and age-matched healthy controls were recruited for this single-center cross-sectional study conducted in Iran, Shiraz, between August 2020 and March 2023. Athletes were selected from sports clubs. Healthy volunteers, who were chosen from the *Imam Reza Clinic* staff, and their relatives or participants who were referred to this clinic for any reason, constituted the control group. All included athletes had undertaken treadmill running for at least 75 min in vigorous intensity per week during the preceding three months or more. They ran on the treadmill once per practice day. Vigorous-intensity activity, defined as metabolic equivalents of tasks (MET) > 6 [2, 16], was determined by achieving a heart rate of 70 to 85% of the maximal heart rate and experiencing labored breathing, indicated by the inability to speak more than a few words without pausing for breath during the talk test [17]. Participants were selected based on a BMI range of 18.5 to 30. They were excluded if they had a systemic disease (e.g., rheumatologic diseases, diabetes mellitus, thyroid disorders, and hormonal imbalances), knee trauma/pain/edema/malalignment or decreased range of motion, inflammatory or infectious arthritis, history of intra-articular injection or knee surgery, previous fracture of the femur, tibia, fibula, or patella, or injury to adjacent structures such as the hamstring or quadriceps muscles, cruciate ligaments, or menisci, as these may influence distal femoral cartilage thickness. Those who directly used supplements containing chondroitin sulfate and glucosamine, known to affect cartilage condition potentially [18], were excluded. The athletes did not participate in other individual or team sports, nor did they run on surfaces other than a treadmill, while the control group had no organized sports activities, including running. According to the 2003–2004 National Health and Nutrition Examination Survey (NHANES) [19], individuals engaged in high or intermediate occupational activity were excluded from the study. The majority of the athletes adhered to a diet typically high in protein, complex carbohydrates, healthy fats, and micronutrient-rich foods. Additionally, their coaches monitored external factors such as sleep hygiene across participants. All cases were informed about the study objectives and procedures and provided written consent to participate. The Institutional Ethics Committee approved the study protocol under the ethical number IR.SUMS.MED.REC.1399.536.

All of the athletes were physique bodybuilders. Physique is a relatively new bodybuilding division, mainly focusing on aesthetically pleasing physiques instead of muscle bulk, with the primary emphasis on upper extremity development [20]. Fitness, muscular shape, symmetry, confidence, and stage presence are other dimensions considered. The athletes engaged in similar targeted upper extremity exercises, such as arm curls, shoulder presses, chest flies, and rows, which are associated with minimal stress and direct loading on the knee. They regularly ran on the treadmills during the week to stay lean, fit, and well-proportioned without participating in other specific lower-extremity sports activities. They were professional athletes actively engaged in rigorous preparation for competitive bodybuilding events and possessed extensive backgrounds in bodybuilding competitions at various levels. Under the diligent supervision of their coach, treadmill running was a meticulously planned component of their training routine. Some athletes may have been new to this aspect of training, while others had more experience, but all stuck to it to enhance their athletic performance.

### Training protocols and data collection

Demographic and clinical features were noted, including age, weight, height, BMI, smoking habits, and occupation, as well as physical activity metrics such as the total months of treadmill running, mean minutes of running per session and week, and mean incline and speed of treadmill running. Athletes didn’t follow a predetermined standardized protocol. However, their training regimens were highly similar due to shared environments, equipment, and athletic goals. Most athletes utilized Sole Fitness treadmills, which are reliable equipment commonly available in fitness facilities, and all used treadmills were shock-absorbent. The intensity of their training, assessed by mean heart rate (HR), mean respiratory rate, distance covered, and calories expended, varied from session to session and week to week according to their established training plan. Each session’s speed, incline, and duration were not exactly uniform and varied depending on the specific training requirements. However, to align with the inclusion criteria of this study, athletes engaged in vigorous-intensity training, as previously defined by MET > 6, for a minimum of 75 min per week, ensuring consistency with recommended guidelines for cardiovascular health [2]. Their coach meticulously planned and supervised their running training regimen to ensure consistency and adherence to specific protocols. Most of the data, including the duration of every training session, mean incline, and speed of each session, were accurately recorded by the coach using treadmill console recordings. Additionally, insights from interviews with athletes provided further details on the perceived normal running routine.

### Cartilage thickness measurement

All measurements were performed using the same US device by a linear probe (7–12 MHz, MyLab™Sigma, Esaote SpA, Genoa, Italy) in the clinic. Measurements were done 3 to 5 days after their last practice session. Participants were asked to sit comfortably on the examination table with their knees in maximum flexion. The physiatrist placed the probe in an axial position on the suprapatellar area. Then, the thickness of the medial (middle part), lateral (middle part), and intra-condylar sections of the distal cartilage of the femur on each side was measured and recorded. The distance between the sharp hyperechoic line at the cartilage-bone interface and the thin hyperechoic line at the synovial space-cartilage interface was measured as the cartilage thickness. The same physician collected all the demographic and clinical data, and a physiatrist expert in the musculoskeletal US performed all the measurements.

Previous studies [21, 22] have validated US as a reliable modality for visualizing femoral cartilage, demonstrating strong agreement between US and MRI measurements and affirming its reliability in assessing cartilage thickness. Similarly, investigations have shown that US measurements of femoral articular cartilage thickness exhibit good reproducibility, characterized by high inter-rater reliability and agreement with anatomical measurements [23, 24]. Several studies have employed this exact method in various populations of patients, indicating its reliability and applicability across different contexts [25, 26]. Although formal inter-rater assessments were not conducted in this study due to consistent measurements by the same sonographer, we ensured measurement consistency and reliability through rigorous adherence to a standardized protocol. The sonographer responsible for data collection underwent thorough training to ensure proficiency in measurement techniques. Additionally, to assess intra-rater reliability, a subset of measurements was randomly selected and independently reviewed by the same sonographer at different time points, demonstrating high consistency and reliability over time.

Specifically, the Intra-class correlation coefficient (ICC) values for the lateral condyle (0.779 [95% CI: 0.707–0.876]), the intercondylar area (0.843 [95% CI: 0.790–0.883]), and the medial condyle (0.834 [95% CI: 0.778–0.835]) were obtained [27].

### Statistical analysis

The required sample size for each group was calculated using G*Power 3.1 software, employing an independent t-test [28]. With a power of 80%, a two-tailed significance level of 5%, and a medium Cohen-suggested effect size of 0.5 [29], a minimum sample size of 64 per group was needed.

The normality of the data was assessed by conducting the Shapiro-Wilk test. An independent t-test was employed to compare cartilage thickness between study groups and between male and female athletes. It was also applied to evaluate other continuous variables such as age, BMI, and minutes of running per session and week between the groups. We conducted an analysis of variance (ANOVA) to compare cartilage thickness between the two different subgroups and controls. Following the ANOVA, a Bonferroni post-hoc test was utilized to evaluate between-group differences. We used the Pearson correlation test to examine the relationship between cartilage thickness and factors like age, weight, height, BMI, treadmill speed, and incline. Spearman correlation was applied to analyze the mean minutes of running per session and week and the total months of activity in relation to cartilage thickness among athletes. The correlation coefficients (r) were interpreted using thresholds commonly used in the literature: very weak (0.000-0.199), weak (0.200-0.399), moderate (0.400-0.599), strong (0.600-0.799), or very strong (0.800-1.000) [30]. We utilized the chi-square test to assess gender distribution among subgroups. Values are presented as mean and standard deviation (SD). All of the analyses were carried out using SPSS software version 26.0 (SPSS Inc., Chicago, Illinois, USA). A P-value of < 0.05 was considered statistically significant.

## Results

### General characteristics

A total of 72 professional athletes with a mean age of 29.6 ± 6.6 years and BMI of 25.9 ± 3.1 kg/m² (52 men, 20 women) and 72 healthy controls with a mean age of 31.9 ± 6.7 years and BMI of 25.6 ± 2.6 kg/m² (52 men, 20 women) were enrolled in this study. The detailed demographic characteristics of the athletes and controls are shown in Table 1. There were no significant differences between the groups regarding age, sex, weight, height, BMI, and smoking status (*P* > 0.05).

**Table 1:** Presents the demographic information and physical activity data of the study participants

Variables	Total Athletes (*n* = 72)	Athletes Subgroup 1 (*n* = 36)	Athletes Subgroup 2 (*n* = 36)	Control Group (*n* = 72)	*P*-value
Age (years), mean ± SD	29.6 ± 6.6	28.4 ± 7.1	30.7 ± 6.0	31.9 ± 6.7	NS
Sex, male/female, n (%)	52 (72%)/20 (28%)	26 (72%)/10 (28%)	26 (72%)/10 (28%)	52 (72%)/20 (28%)	NS
Weight (kg), mean ± SD	79.6 ± 12.9	77.8 ± 11.7	81.5 ± 14.0	77.5 ± 10.0	NS
Height (cm), mean ± SD	170.1 ± 30.2	168.9 ± 30.1	171.3 ± 30.7	171.7 ± 27.9	NS
BMI (kg/m^2^), mean ± SD	25.9 ± 3.1	25.6 ± 3.2	26.1 ± 3.0	25.6 ± 2.6	NS
Smokers, n (%)	11 (15.2)	6 (16.6)	5 (13.8)	14 (19.4)	NS
Speed (mph), mean ± SD	8.7 ± 2.8	8.2 ± 2.3	9.3 ± 3.1	-	NS
Incline (degree), mean ± SD	2.6 ± 3.4	2.2 ± 2.8	3.1 ± 3.9	-	NS
Mean running duration per session (minutes), mean ± SD	31.4 ± 5.1	32.6 ± 5.3	30.1 ± 4.3	-	NS
Mean running duration per week (minutes), mean ± SD	108.7 ± 48.6	109.3 ± 49.8	108.4 ± 48.0	-	NS
Total duration of running (months), mean ± SD	17.2 ± 15.9	5.1 ± 1.8	29.3 ± 14.5	-	**0.001**

SD: standard deviation, BMI: body mass index, NS: not significant, kg: kilogram, cm: centimeter, mph: mile per hour. Subgroup 1: athletes with less than 12 months running, subgroup 2: athletes with equal or more than 12 months running

### Cartilage thickness comparison between all athletes and controls

Table 2 shows the mean distal femoral cartilage thickness between all athletes and controls. Although the athletes had thinner femoral cartilage thickness values at all measured sites compared with the controls, the difference only reached statistical significance in the RLC (2.21 ± 0.38 cm vs. 2.39 ± 0.31 cm, *p* = 0.002), LLC (2.28 ± 0.37 cm vs. 2.46 ± 0.35 cm, *p* = 0.004), and LMC (2.28 ± 0.42 cm vs. 2.42 ± 0.36 cm, *p* = 0.039).

**Table 2:** Distal Femoral Cartilage Thickness Measurements of Participants

Mean thickness/ SD (cm)	Total Athletes	Control	*P*-value
RLC	2.21 ± 0.38	2.39 ± 0.31	0.002*
RIA	2.31 ± 0.50	2.42 ± 0.48	0.170
RMC	2.30 ± 0.39	2.38 ± 0.38	0.201
LLC	2.28 ± 0.37	2.46 ± 0.35	0.004*
LIA	2.33 ± 0.54	2.46 ± 0.48	0.077
LMC	2.28 ± 0.42	2.42 ± 0.36	0.039*

RLC: Right Lateral Condyle, RIA: Right Intercondylar Area, RMC: Right Medial Condyle, LLC: Left Lateral Condyle, LIA: Left Intercondylar Area, LMC: Left Medial Condyle). * Independent t-test was used to compare cartilage thickness in total athletes and controls

**Fig. 1:**
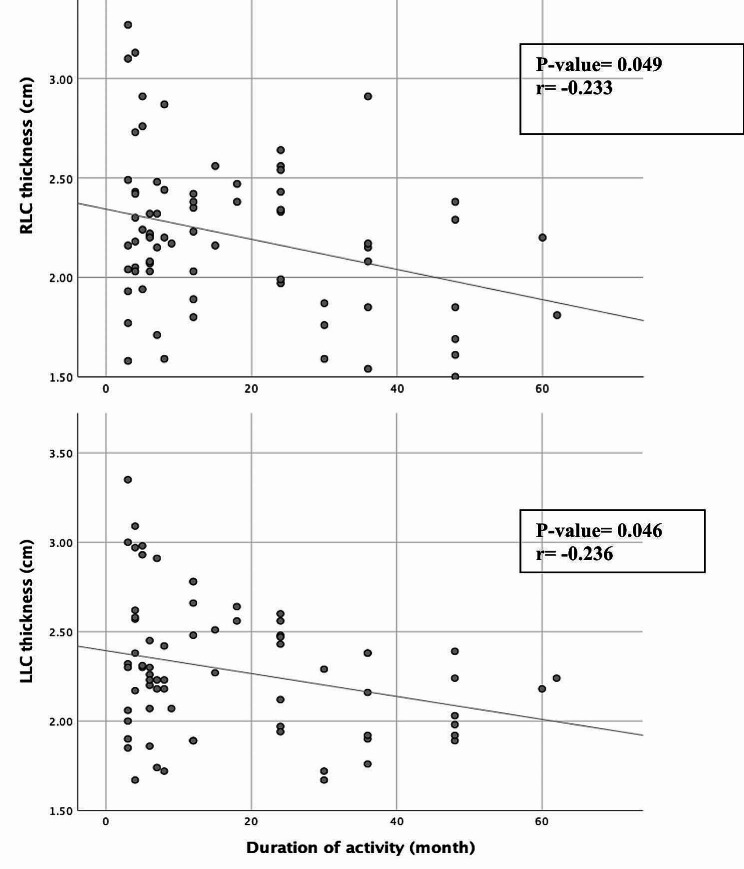
Scatter plot depicting the correlation between duration of running and cartilage thickness. RLC: Right Lateral Condyle, LLC: Left Lateral Condyle, r: Correlation coefficient

There was a weak, significant negative correlation between the duration of treadmill running and distal femoral cartilage thickness in the RLC (*r* = -0.236, *p* = 0.046) and LLC (*r* = -0.233, *p* = 0.049), indicating that as the duration of treadmill running increases, the thickness of cartilage decreases (Fig. 1). However, no significant correlation was found between the distal femoral cartilage thickness at different sites and the athletes’ age, BMI, speed and incline of treadmill running, and minutes of running per session and week (*p* > 0.05). Female athletes had significantly thinner distal femoral cartilages in RLC (2.29 ± 0.37 cm vs. 1.98 ± 0.33, *p* = 0.002), RIC (2.42 ± 0.49 cm vs. 2.02 ± 0.40 cm, *p* = 0.002), LLC (2.36 ± 0.36 cm vs. 2.06 ± 0.30 cm, *p* = 0.002) and, LMC (2.34 ± 0.042 cm vs. 0.14 ± 0.40 cm, *p* = 0.008) than male athletes.

### Subgroup analyzing

**Fig. 2:**
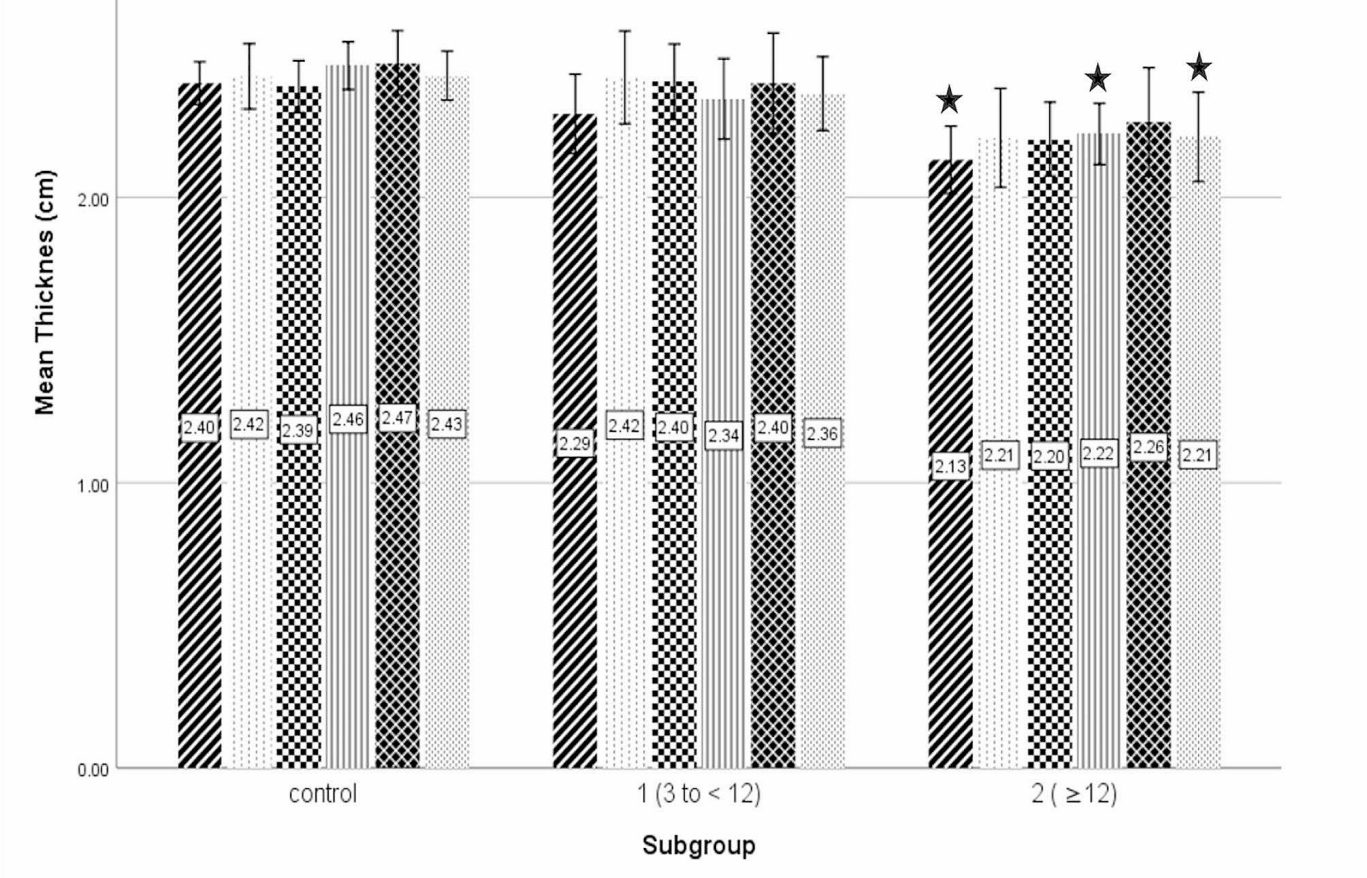
The mean distal femoral cartilage thickness in various regions of the knees within subgroups is depicted at the center of each bar. RLC: right lateral condyle, RIA: right intercondylar area, RMC: right medial condyle, LMC: left medial condyle, LIA: left intercondylar area, LLC: left lateral condyle; Subgroup 1: 3 to < 12 months of treadmill running, Subgroup 2: ≥12 months of treadmill running

Table 1 shows demographic comparisons between subgroup 1, 2 and controls. To examine how different durations of running, including short-term (less than 12 months) and longer-term durations (12 months or more) could influence cartilage thickness, a subgroup analysis was performed. Subgroup 1 consisted of 36 athletes, (26 men and 10 women) with a mean age of 28.4 ± 7.1 years, and a mean duration of treadmill running of 5.1 ± 1.8 months. Subgroup 2 also comprised 36 athletes (26 men and 10 women) with a mean age of 30.7 ± 6.0 years and, a mean duration of treadmill running of 29.3 ± 14.5 months (Table 1). The mean cartilage thickness for each subgroup is provided in Fig. 2. The analysis revealed that there were no significant gender distribution differences among the subgroups (*p* > 0.05). When comparing the subgroups of athletes with the controls, the analysis showed significantly thinner cartilage thickness in the RLC (2.13 ± 0.34 cm vs. 2.39 ± 0.31 cm, *p* = 0.001), LLC (2.22 ± 0.31 cm vs. 2.46 ± 0.35 cm, *p* = 0.005), and LMC (2.21 ± 0.46 cm vs. 2.42 ± 0.36, cm, *p* = 0.027) in subgroup 2 (≥ 12 months of treadmill running) compared with the controls (Fig. 2). No significant differences were observed in cartilage thickness between subgroup 1 and 2, or between subgroup 1 and the control group.

## Discussion

The aims of this study were to investigate the effects of treadmill running on knee cartilage thickness in professional athletes compared with non-athlete individuals, by using US, and to determine whether there is a correlation between the total months of treadmill running and cartilage thickness in various knee regions. One main finding of this study was that athletes with over one year (mean 29, SD 14.5 months) of treadmill running experience had significantly thinner cartilage thickness in the majority of knee regions compared with non-athlete controls. Another finding was that a weak negative correlation between the total months of treadmill running and distal femoral cartilage thickness in different knee regions among physique bodybuilders was revealed. No significant correlations were observed between cartilage thickness and treadmill incline, speed, BMI, or mean duration of minutes of running per session and week.

Prior studies have also examined the acute effects of treadmill running on femoral cartilage thickness. Güvener et al. found no significant difference in absolute and percent change of cartilage thickness following one session of treadmill jogging compared with walking and resting in healthy participants [31]. Conversely, Harkey et al. reported significant medial knee cartilage deformation in healthy participants during treadmill running and walking compared with rest [32]. The present study primarily focuses on the long-term effects of treadmill running rather than immediate changes after a single session, and it indicates a weak negative correlation between total months of treadmill running and cartilage thickness. It’s important to note that while acute changes in cartilage thickness are often reversible, long-term participation in treadmill running may lead to permanent alterations, which is beyond articular cartilage adaption capacity [33]. From a molecular perspective, this chronic process involves various changes in cartilage structure, including chondrocyte hypertrophy, cartilage zone clustering, small fissures in the cartilage, increased expression of the aggrecan gene, reduced expression of matrix metallopeptidase-2 (MMP-2) and mono-iodoacetate, loss of glycosaminoglycans (GAGs), and resorption of periarticular bone [34–37].

The increasing global popularity of motorized treadmills spans various settings, including sports clubs, gyms, medical facilities, and home users. About 14% of American runners and elite athletes utilize motorized treadmills as part of their training routine [3].

In this study, only athletes with high-intensity running training were included and were shown to have less cartilage thickness, especially those with longer experience of running, compared with controls. Intense physical activities, such as treadmill running, exert significant stress on articular cartilage, potentially leading to cartilage strains and reduced thickness in the tibiofemoral joint [38, 39]. While moderate-intensity running may help maintain cartilage integrity, high-intensity treadmill running can alter the subchondral bone composition, making it stiff and fragile, thus increasing the mechanical load on overlying cartilage and promoting degeneration [40, 41]. Prior research predominantly explores the impact of high-intensity treadmill running on articular cartilage using animal models. Kotwal et al. observed significant decreases in cartilage thickness and volume in mice subjected to excessive treadmill running, suggesting a role of proteoglycan (PG) loss in these changes [42]. A four-week high-intensity treadmill regimen also induced irreversible cartilage catabolism and weakened anabolism in mice [43]. Tang et al. demonstrated femoral articular cartilage degeneration in rats following six weeks of strenuous treadmill running [44]. Understanding the impact of activity intensity is vital, as higher weight-bearing loads can render articular cartilage more susceptible to degenerative changes [5, 45, 46].

Although this study does not directly measure the distance covered by athletes, there was no difference between subgroup 1 and 2 in either minutes per session or week of running or running speed. However, there was a difference between subgroup 2 and controls regarding the thickness of cartilage, yet not between subgroup 1 and controls, implying that it may be the history of running that might affect this. This aligns with the notion that prolonged participation in high-intensity activities, such as long-distance running, may impact knee joint health over time [47]. Therefore, while this study does not assess the exact distance covered, it provides insights into the potential effects of sustained treadmill running on knee cartilage thickness, especially over a year. The Hinterwimmer study noted significant cartilage loss in the lateral femoral cartilage after six months of a training program followed by a marathon, particularly among marathon beginners [48]. Sohn and Micheli investigated former cross-country runners, contrasting them with swimmers as a control group, yet found no discernible link between long-distance running and the development of OA [49]. Chakravarty et al. tracked knee OA progression over two decades in middle-to-older-aged long-distance runners versus healthy non-runners [50]. Despite assessing 55 long-distance runners and 53 controls with a mean age of 58 years, they found no increased radiologic OA progression in long-distance runners. The inconsistency of these results with the present study may stem from differences in running surfaces, as treadmill running differs from ground running in various aspects. A systematic review by Semaan et al. highlights that while motorized treadmill running and overground running generally yield comparable outcomes across measures like spatiotemporal, kinematic, kinetic, electromyographic, and energy consumption, disparities emerge in certain aspects such as kinematics, kinetics, ground reaction force components, and electromyographic responses, particularly with long-term, high-intensity usage [51, 52]. Also, some animal studies have reported positive effects of treadmill running on knee cartilage health [40, 41, 45, 46]. However, disparities in methodology, excluding the inherent differences due to animal nature, may contribute to variations in results compared with this study. Nonetheless, accurately predicting the effect of treadmill running on knee cartilage remains challenging.

This study investigated various parameters, including incline running, treadmill speed, BMI, gender, and side differences, in relation to knee cartilage thickness. The findings largely align with prior research, with some distinctions noted. Regarding treadmill speed, the present study found no significant impact on knee cartilage thickness, consistent with the findings of Rios et al. [53]. We also observed gender differences in cartilage thickness, consistent with previous researches, where female athletes tended to have thinner cartilage compared with males [54, 55]. While no correlation was found between incline running on a treadmill and cartilage thickness in this study, it is suggested that incline walking may positively affect cartilage degeneration by increasing abduction knee moment [56]. We found no significant correlation between BMI and cartilage thickness, contrary to studies suggesting a link between higher BMI and thinner cartilage [53, 57]. Notably, all athletes in this study had a BMI under 30 kg/m2. It is also indicated that individuals with higher BMI (> 30 kg/m2) showed increased cartilage strain during walking compared with those with normal BMI, suggesting the possibility that the meniscus may not effectively distribute loads to mitigate the effects of obesity [57]. Furthermore, this study did not find discrepancies in cartilage thickness between the left and right distal femoral sides, contrary to the study by Adam et al. [58], which found that the right lateral side was thicker than the left, possibly due to most people being right-footed.

Interestingly, the present study found no correlation between daily minutes of treadmill running and cartilage thickness. Research suggests that moderate daily exercise may benefit cartilage matrix composition, but not thickness, in healthy animals, while high exercise doses could have negative effects [59]. Notably, shorter durations with more frequent sessions of treadmill activity per day may have chondroprotective effects, as demonstrated by Yang et al., where 60 min of daily treadmill running completed in three sessions was found to be more beneficial for cartilage health compared with fewer sessions with longer durations [60]. These disparities may suggest the need for further investigation into the complex relationship between these parameters and knee cartilage health. It is attempting to assume that degenerative articular cartilage changes may appear when high-intensity treadmill running continues for a long-term of months, especially in weight-bearing femoral cartilage among physique bodybuilders. High intensity and overuse are responsible in this regard, as physique bodybuilders with more than one year of vigorous-intensity treadmill running had significantly thinner distal femoral cartilages in both knees’ lateral and medial sides.

### Limitations

Relying solely on a single methodological approach may limit the depth of this study’s analysis. Incorporating multi-modal research methods could offer a more comprehensive understanding of the phenomenon under investigation. For example, histological and molecular evaluations using multiple imaging modalities may improve understanding of the topic and help to explore the underlying mechanisms sufficiently. However, we must consider such assessments’ ethical issues, costs, and challenges when dealing with human participants. In addition, despite the efforts to apply strict inclusion and exclusion criteria, controlling all variables is challenging. For example, treadmill devices may have different shock absorbent capabilities. It’s also crucial to highlight the potential impact of other possible limited lower extremity physical activity and the indirect effects of upper extremity activity on knee cartilage through biomechanical and systemic responses. However, such impacts seem minimal and inconclusive. Most importantly, the present study was retrospective. Although an attempt to utilize documented information with the assistance of their coaches while collecting information regarding athletes’ physical activity was performed, it is important to acknowledge that the possibility of recall bias cannot be completely excluded. so future longitudinal studies with prolonged follow-ups are necessary to fix the limitations and draw cause-and-effect conclusions between vigorous-intensity treadmill running and cartilage thickness changes.

## Conclusion

According to the findings of this study, we conclude that vigorous sustained treadmill running may have adverse effects on knee cartilage in athletes, specifically those with more than a year of running, despite the suggested priority of a more physically active lifestyle compared with a sedentary one. Duration of treadmill running, rather than incline and, speed of running, age, and BMI, was negatively correlated with distal femoral cartilage thickness. To optimize training and prevent adverse effects, identifying the conditions under which they occur is crucial. Factors such as intensity, frequency of training, type of physical activity, and environmental variables like treadmill settings play significant roles in this optimization process.

### Electronic supplementary material

Below is the link to the electronic supplementary material.

Supplementary Material 1

## Data Availability

The datasets utilized and/or analyzed during the present study are accessible upon reasonable request from the corresponding author.

## Abbreviations

RLC: Right lateral condyle
RIA: Right intercondylar area
RMC: Right medial condyle
LLC: Left lateral condyle
LIA: Left intercondylar area
LMC: Left medial condyle
BMI: Body mass index
OA: Osteoarthritis
SD: Standard deviation
MMP-2: Matrix metallopeptidase-2
GAG: Glycosaminoglycan
PG: Proteoglycan
ICC: Intra-class correlation coefficient
US: Ultrasonography

## References

[1] Fillon A, Genin P, Larras B, Vanhelst J, Luiggi M, Aubert S (2021). France’s 2020 report card on physical activity and sedentary behaviors in children and youth: results and progression. J Phys Activity Health.

[2] Piercy KL, Troiano RP, Ballard RM, Carlson SA, Fulton JE, Galuska DA (2018). The physical activity guidelines for americans. JAMA.

[3] Van Hooren B, Fuller JT, Buckley JD, Miller JR, Sewell K, Rao G (2020). Is Motorized Treadmill running biomechanically comparable to Overground running? A systematic review and Meta-analysis of Cross-over studies. Sports Med.

[4] Gunter K, Baxter-Jones A, Mirwald R, Almstedt H, Fuchs R, Durski S, Snow C (2008). Impact Exercise increases BMC during growth: an 8-Year longitudinal study. J bone Mineral Research: Official J Am Soc Bone Mineral Res.

[5] Gao J, Fang J, Gong H, Gao B. Morphological and microstructural alterations of the articular cartilage and bones during treadmill exercises with different additional weight-bearing levels. Journal of Healthcare Engineering. 2017;2017. 10.1155/2017/8696921 PMC5525086 29065659

[6] Beckett J, Jin W, Schultz M, Chen A, Tolbert D, Moed BR, Zhang Z (2012). Excessive running induces cartilage degeneration in knee joints and alters gait of rats. J Orthop Res.

[7] Murray R, Duncan C (1971). Athletic activity in adolescence as an etiological factor in degenerative hip disease. J bone Joint Surg Br Volume.

[8] Cymet TC, Sinkov V (2006). Does long-distance running cause osteoarthritis?. J Am Osteopath Assoc.

[9] Kujala UM, Kaprio J, Sarno S (1994). Osteoarthritis of weight bearing joints of lower limbs in former elite male athletes. BMJ.

[10] Lu L, Wang Y (2014). Effects of exercises on knee cartilage volume in young healthy adults: a randomized controlled trial. Chin Med J (Engl).

[11] Khan MCM, O’Donovan J, Charlton JM, Roy JS, Hunt MA, Esculier JF (2022). The influence of running on lower limb cartilage: a systematic review and Meta-analysis. Sports Med.

[12] Coburn SL, Crossley KM, Kemp JL, Warden SJ, West TJ, Bruder AM (2023). Is running good or bad for your knees? A systematic review and meta-analysis of cartilage morphology and composition changes in the tibiofemoral and patellofemoral joints. Osteoarthritis Cartilage.

[13] Eckstein F, Hudelmaier M, Putz R (2006). The effects of exercise on human articular cartilage. J Anat.

[14] Trovato B, Petrigna L, Sortino M, Roggio F, Musumeci G (2023). The influence of different sports on cartilage adaptations: a systematic review. Heliyon.

[15] von Elm E, Altman DG, Egger M, Pocock SJ, Gøtzsche PC, Vandenbroucke JP. The Strengthening the Reporting of Observational Studies in Epidemiology (STROBE) statement: guidelines for reporting observational studies. J Clin Epidemiol. 2008;61(4):344–9. 10.1016/j.jclinepi.2007.11.008 18313558

[16] Verschuren O, Mead G, Visser-Meily A (2015). Sedentary behaviour and stroke: foundational knowledge is crucial. Translational Stroke Res.

[17] Division of Nutrition PA, and Obesity, National Center for Chronic Disease Prevention and Health Promotion. Measuring Physical Activity Intensity 2022 [ https://www.cdc.gov/physicalactivity/basics/measuring/index.html#print.

[18] Henrotin Y, Marty M, Mobasheri A (2014). What is the current status of chondroitin sulfate and glucosamine for the treatment of knee osteoarthritis?. Maturitas.

[19] Steeves JA, Tudor-Locke C, Murphy RA, King GA, Fitzhugh EC, Harris TB (2015). Classification of occupational activity categories using accelerometry: NHANES 2003–2004. Int J Behav Nutr Phys Activity.

[20] Roberts BM, Helms ER, Trexler ET, Fitschen PJ (2020). Nutritional recommendations for physique athletes. J Hum Kinetics.

[21] Kauppinen K, Casula V, Zbýň Š, Blanco Sequeiros R, Saarakkala SS, Nevalainen MT. Ultrasonographic Assessment of the Normal Femoral Articular Cartilage of the Knee Joint: Comparison with 3D MRI. ScientificWorldJournal. 2021;2021:9978819. 10.1155/2021/9978819 PMC8387170 34456636

[22] Pradsgaard D, Fiirgaard B, Spannow AH, Heuck C, Herlin T (2015). Cartilage thickness of the knee joint in juvenile idiopathic arthritis: comparative assessment by ultrasonography and magnetic resonance imaging. J Rheumatol.

[23] Naredo E, Acebes C, Möller I, Canillas F, de Agustín JJ, de Miguel E (2009). Ultrasound validity in the measurement of knee cartilage thickness. Ann Rheum Dis.

[24] Yoon CH, Kim HS, Ju JH, Jee WH, Park SH, Kim HY (2008). Validity of the sonographic longitudinal sagittal image for assessment of the cartilage thickness in the knee osteoarthritis. Clin Rheumatol.

[25] Kesikburun S, Köroglu Ö, Yasar E, Güzelküçük Ü, Yazcoglu K, Tan AK (2015). Comparison of intact knee cartilage thickness in patients with traumatic lower extremity amputation and nonimpaired individuals. Am J Phys Med Rehabil.

[26] Yildizgoren MT, Helvaci MR, Ustun N, Osmanoglu K, Turhanoglu AD (2016). Ultrasonographic assessment of the distal femoral cartilage thickness in patients with homozygous sickle cell disease. Cartilage.

[27] Roberts HM, Moore JP, Thom JM (2019). The reliability of suprapatellar transverse sonographic assessment of femoral trochlear cartilage thickness in healthy adults. J Ultrasound Med.

[28] Kang H. Sample size determination and power analysis using the G* power software. J Educational Evaluation Health Professions. 2021;18. 10.3352/jeehp.2021.18.17 PMC8441096 34325496

[29] Sullivan GM, Feinn R (2012). Using effect size—or why the P value is not enough. J Graduate Med Educ.

[30] Care F, Sugeng Subagio B, Rahman H, Ridwan Aldila Melania (2018). Porous concrete basic property criteria as rigid pavement base layer in Indonesia. MATEC Web Conf.

[31] Güvener O, Dağ F, Çimen ÖB, Özçakar L (2022). Ultrasound assessment of distal femoral cartilage thickness measurements after walking/jogging in subjects with pes planus. Knee.

[32] Harkey M, Blackburn J, Davis H, Sierra-Arevalo L, Nissman D, Pietrosimone B (2017). Ultrasonographic assessment of medial femoral cartilage deformation acutely following walking and running. Osteoarthr Cartil.

[33] Bini RR, Bini AF (2020). Effects of exercise mode in knee cartilage thickness. J Bodyw Mov Ther.

[34] Moshtagh PR, Korthagen NM, Plomp SG, Pouran B, Castelein RM, Zadpoor AA, Weinans H (2018). Early signs of bone and cartilage changes induced by treadmill exercise in rats. JBMR plus.

[35] Miyatake K, Muneta T, Ojima M, Yamada J, Matsukura Y, Abula K (2016). Coordinate and synergistic effects of extensive treadmill exercise and ovariectomy on articular cartilage degeneration. BMC Musculoskelet Disord.

[36] Ni G-X, Liu S-Y, Lei L, Li Z, Zhou Y-Z, Zhan L-Q. Intensity-dependent effect of treadmill running on knee articular cartilage in a rat model. BioMed Research International. 2013;2013. 10.1155/2013/172392 PMC3892754 24693534

[37] Saito R, Muneta T, Ozeki N, Nakagawa Y, Udo M, Yanagisawa K (2017). Strenuous running exacerbates knee cartilage erosion induced by low amount of mono-iodoacetate in rats. BMC Musculoskelet Disord.

[38] Sutter EG, Widmyer MR, Utturkar GM, Spritzer CE, Garrett WE, DeFrate LE (2015). In vivo measurement of localized tibiofemoral cartilage strains in response to dynamic activity. Am J Sports Med.

[39] Lad NK, Liu B, Ganapathy PK, Utturkar GM, Sutter EG, Moorman CT (2016). Effect of normal gait on in vivo tibiofemoral cartilage strains. J Biomech.

[40] Li Z, Liu S-Y, Xu L, Xu S-Y, Ni G-X (2017). Effects of treadmill running with different intensity on rat subchondral bone. Sci Rep.

[41] Day J, Ding M, Van Der Linden J, Hvid I, Sumner D, Weinans H (2001). A decreased subchondral trabecular bone tissue elastic modulus is associated with pre-arthritic cartilage damage. J Orthop Res.

[42] Kotwal N, Li J, Sandy J, Plaas A, Sumner DR (2012). Initial application of EPIC-µCT to assess mouse articular cartilage morphology and composition: effects of aging and treadmill running. Osteoarthr Cartil.

[43] Zhou X, Cao H, Wang M, Zou J, Wu W (2021). Moderate-intensity treadmill running relieves motion-induced post-traumatic osteoarthritis mice by up-regulating the expression of lncRNA H19. Biomed Eng Online.

[44] Tang T, Muneta T, Ju Y-J, Nimura A, Miyazaki K, Masuda H (2008). Serum keratan sulfate transiently increases in the early stage of osteoarthritis during strenuous running of rats: protective effect of intraarticular hyaluronan injection. Arthritis Res Therapy.

[45] Iijima H, Aoyama T, Ito A, Tajino J, Yamaguchi S, Nagai M (2016). Exercise intervention increases expression of bone morphogenetic proteins and prevents the progression of cartilage-subchondral bone lesions in a post-traumatic rat knee model. Osteoarthr Cartil.

[46] Stehling C, Lane NE, Nevitt MC, Lynch J, McCulloch CE, Link TM (2010). Subjects with higher physical activity levels have more severe focal knee lesions diagnosed with 3 T MRI: analysis of a non-symptomatic cohort of the osteoarthritis initiative. Osteoarthr Cartil.

[47] Timmins KA, Leech RD, Batt ME, Edwards KL (2017). Running and knee osteoarthritis: a systematic review and meta-analysis. Am J Sports Med.

[48] Hinterwimmer S, Feucht MJ, Steinbrech C, Graichen H, von Eisenhart-Rothe R (2014). The effect of a six-month training program followed by a marathon run on knee joint cartilage volume and thickness in marathon beginners. Knee Surg Sports Traumatol Arthrosc.

[49] SOHN RS, MICHELI LJ (1985). The effect of running on the pathogenesis of osteoarthritis of the hips and knees. Clin Orthop Relat Research®.

[50] Chakravarty EF, Hubert HB, Lingala VB, Zatarain E, Fries JF (2008). Long distance running and knee osteoarthritis: a prospective study. Am J Prev Med.

[51] Semaan MB, Wallard L, Ruiz V, Gillet C, Leteneur S, Simoneau-Buessinger E (2022). Is treadmill walking biomechanically comparable to overground walking? A systematic review. Gait Posture.

[52] AlGheshyan F, Eltoukhy M, Onar A-T, Asfour S (2015). Development of a regres-sion model for the treadmill ground reaction force components. Sport Ex-erc Med Open J.

[53] Rios JL, Boldt KR, Mather JW, Seerattan RA, Hart DA, Herzog W (2018). Quantifying the effects of different treadmill training speeds and durations on the health of rat knee joints. Sports Medicine-Open.

[54] Brenneman Wilson EC, Gatti AA, Maly MR. A new technique to evaluate the impact of running on knee cartilage deformation by region. Magn Reson Mater Phys Biol Med. 2021:1–11. 10.1007/s10334-020-00896-8 33387105

[55] Özçakar L, Tunc H, Öken Ö, Ünlü Z, Durmuş B, Baysal Ö (2014). Femoral cartilage thickness measurements in healthy individuals: learning, practicing and publishing with TURK-MUSCULUS. J Back Musculoskelet Rehabil.

[56] Haggerty M, Dickin DC, Popp J, Wang H (2014). The influence of incline walking on joint mechanics. Gait Posture.

[57] Collins AT, Kulvaranon M, Spritzer CE, McNulty AL, DeFrate LE (2020). The influence of obesity and Meniscal Coverage on in vivo tibial cartilage thickness and strain. Orthop J Sports Med.

[58] ADAM C, ECKSTEIN F, PUTZ MILZS (1998). The distribution of cartilage thickness within the joints of the lower limb of elderly individuals. J Anat.

[59] Bricca A, Juhl C, Grodzinsky A, Roos E (2017). Impact of a daily exercise dose on knee joint cartilage–a systematic review and meta-analysis of randomized controlled trials in healthy animals. Osteoarthr Cartil.

[60] Yang Y, Wang Y, Kong Y, Zhang X, Bai L (2017). The effects of different frequency treadmill exercise on lipoxin A4 and articular cartilage degeneration in an experimental model of monosodium iodoacetate-induced osteoarthritis in rats. PLoS ONE.

