# Supplementary material for: Impact of treadmill running on distal femoral cartilage thickness: a cross-sectional study of professional athletes and healthy controls
Source: BMC Sports Sci Med Rehabil. 2024 May 6;16:104. doi: 10.1186/s13102-024-00896-4 (PMC11071246; doi:10.1186/s13102-024-00896-4)
Supplement: Supplementary file 1 — Supplementary Material 1 [file 13102_2024_896_MOESM1_ESM.docx]

STROBE Statement—checklist of items that should be included in reports of observational studies

|  | Item No. | Recommendation | Page  No. | Relevant text from manuscript |
| --- | --- | --- | --- | --- |
| **Title and abstract** | 1 | (*a*) Indicate the study’s design with a commonly used term in the title or the abstract | 1 | Impact of treadmill running on distal femoral cartilage thickness: A cross-sectional study of professional athletes and healthy controls |
|  |  | (*b*) Provide in the abstract an informative and balanced summary of what was done and what was found | 2 | This present study aimed to assess the impact of treadmill running on distal femoral cartilage thickness.  Compared to healthy controls, professional athletes with a history of high-intensity treadmill running had thinner femoral cartilages. |
| Introduction | | | |  |
| Background/rationale | 2 | Explain the scientific background and rationale for the investigation being reported | 4 | A sedentary lifestyle and obesity can lead to osteoarthritis, while moderate exercise can prevent this condition. However, when it comes to vigorous physical activity performed by elite or professional athletes, hazards can be expected. |
| Objectives | 3 | State specific objectives, including any prespecified hypotheses | 5 | The long-term impact of sustained treadmill running on joint health remains uncertain. To address this gap in knowledge, we aimed to evaluate the distal femoral cartilage thickness among professional athletes engaged in treadmill running using ultrasonography. Consequently, this study aims to investigate the effect of high-intensity treadmill running on distal femoral cartilage thickness and explore potential factors contributing to cartilage thickness loss. |
| Methods | | | |  |
| Study design | 4 | Present key elements of study design early in the paper | 6 | In this study, we aimed to investigate the differences in cartilage thickness between athletes aged 20-40 years with a minimum of three months of treadmill running, engaging in high-intensity sessions of at least 75 minutes per week. Participants were recruited from various sport clubs, while healthy control subjects were selected from the staff of Imam Reza Clinic. The study design emphasized the recruitment of physically active individuals to examine the potential impact of regular treadmill running on cartilage thickness, utilizing a comparative approach between athletes and healthy controls. |
| Setting | 5 | Describe the setting, locations, and relevant dates, including periods of recruitment, exposure, follow-up, and data collection | 6 | Professional athletes aged 20-40 and age-matched healthy controls were recruited for this single-center cross-sectional study conducted in Iran, Shiraz, between August 2020 and March 2023. |
| Participants | 6 | (*a*) *Cohort study*—Give the eligibility criteria, and the sources and methods of selection of participants. Describe methods of follow-up  *Case-control study*—Give the eligibility criteria, and the sources and methods of case ascertainment and control selection. Give the rationale for the choice of cases and controls  *Cross-sectional study*—Give the eligibility criteria, and the sources and methods of selection of participants | 6 | All included athletes had undertaken treadmill running for at least 75 minutes in vigorous intensity per week during the preceding three months or more. They ran on the treadmill once per practice day. Vigorous-intensity activity, defined as metabolic equivalents of tasks (MET) > 6 (2, 18), was determined by achieving a heart rate of 70 to 85% of the maximal heart rate and experiencing labored breathing, indicated by the inability to speak more than a few words without pausing for breath during the talk test (19). Participants were selected based on a BMI range of 18.5 to 30. They were excluded if they had a systemic disease (e.g., rheumatologic diseases, diabetes mellitus, thyroid disorders, and hormonal imbalances), knee trauma/pain/edema/malalignment or decreased range of motion, inflammatory or infectious arthritis, history of intra-articular injection or knee surgery, previous fracture of the femur, tibia, fibula, or patella, or injury to adjacent structures such as the hamstring or quadriceps muscles, cruciate ligaments, or menisci, as these may influence distal femoral cartilage thickness. Those directly using supplements containing Chondroitin sulfate and glucosamine—known to affect cartilage condition potentially—were excluded. The athletes did not participate in other individual or team sports, nor did they run on surfaces other than a treadmill, while the control group had no organized sports activities, including running. According to the 2003–2004 National Health and Nutrition Examination Survey (NHANES) (20), individuals engaged in high or intermediate occupational activity were excluded from the study. It's worth noting that most participants adhered to a diet typically high in protein, complex carbohydrates, healthy fats, and micronutrient-rich foods. Additionally, their coaches monitored external factors such as sleep hygiene across participants. Athletes were selected from sports clubs. |
|  |  | (*b*) *Cohort study*—For matched studies, give matching criteria and number of exposed and unexposed  *Case-control study*—For matched studies, give matching criteria and the number of controls per case | 6 | Healthy volunteers constitute the control group. the control group had no organized sports activities, including running. The study employed a 1:1 case-control ratio. |
| Variables | 7 | Clearly define all outcomes, exposures, predictors, potential confounders, and effect modifiers. Give diagnostic criteria, if applicable | 6-8 | **Outcomes**: Distal femoral cartilage thickness. **Exposures/Predictors**: Treadmill running. **Treadmill Parameters**: Incline and speed. **Potential Confounders**: Adjusted for age, BMI, running frequency/duration, previous injuries, and other relevant factors. **Effect Modifiers**: Consider age, gender, and other factors influencing treadmill running's impact on cartilage thickness, such as incline and speed of treadmill running. **Diagnostic Criteria**: **Professional Athletes**: Individuals with a history of competing in physique bodybuilding competitions and achieving victories. **Control Group**: The control group consisted of Imam Reza Clinic staff and their relatives or participants without a history of professional athletic competition. |
| Data sources/ measurement | 8* | For each variable of interest, give sources of data and details of methods of assessment (measurement). Describe comparability of assessment methods if there is more than one group | 8 | All measurements were performed using the same ultrasonography device by a linear probe (7-12 MHz, MyLab™Sigma, Esaote SpA, Genoa, Italy) in the clinic. Measurements were done 3 to 5 days after their last practice session. Participants were asked to sit comfortably on the examination table with their knees in maximum flexion. The physiatrist placed the probe in an axial position on the suprapatellar area. Then, the thickness of the medial (middle part), lateral (middle part), and intra-condylar sections of the distal cartilage of the femur on each side was measured and recorded. The distance between the sharp hyperechoic line at the cartilage-bone interface and the thin hyperechoic line at the synovial space-cartilage interface was measured as the cartilage thickness. The same physician collected all the demographic and clinical data, and a physiatrist expert in musculoskeletal ultrasonography performed all the measurements. |
| Bias | 9 | Describe any efforts to address potential sources of bias | 7, 15 | Their coach meticulously planned and supervised their running training regimen to ensure consistency and adherence to specific protocols. Most of the data, including the duration of every training session, mean incline, and speed of each session, were accurately recorded by the coach using treadmill console recordings. Additionally, insights from interviews with athletes provided further details on the perceived normal running routine.  In addition, despite our efforts to apply strict inclusion and exclusion criteria, controlling all variables is challenging. |
| Study size | 10 | Explain how the study size was arrived at | 7 | The required sample size for each group was calculated using G*Power 3.1 software, employing an independent t-test (23). With a power of 80%, a two-tailed significance level of 5%, and a medium Cohen-suggested effect size of 0.5 (24), a minimum sample size of 64 per group was needed. |

Continued on next page

| Quantitative variables | 11 | Explain how quantitative variables were handled in the analyses. If applicable, describe which groupings were chosen and why | 10 | Quantitative variables were handled by categorizing participants based on their duration of activity: more than one year and less than one year. This grouping allowed us to explore differences in cartilage thickness based on sustained activity duration. It was chosen to investigate the potential impact of long-term treadmill running on cartilage health and enabled straightforward comparison between the two groups. |
| --- | --- | --- | --- | --- |
| Statistical methods | 12 | (*a*) Describe all statistical methods, including those used to control for confounding | 9 | The normality of our data was assessed by conducting the Shapiro-Wilk test. An independent t-test was employed to compare cartilage thickness between study groups and among male and female athletes. It was also applied to evaluate other continuous variables such as age, BMI, and minutes of activity per session and week among the groups. We conducted an analysis of variance (ANOVA) to compare cartilage thickness among different subgroups and controls. Following the ANOVA, we utilized the Bonferroni test as the post-hoc test to evaluate between-group differences. We used the Pearson correlation test to examine the relationship between cartilage thickness and factors like age, weight, height, BMI, treadmill speed, and incline. Spearman correlation was applied to analyze the mean duration of activity per session and week and the total months of activity in relation to cartilage thickness among athletes. The correlation coefficients (r) were interpreted using thresholds commonly used in the literature: weak (0.00-0.30), moderate (0.30-0.50), strong (0.50-0.70), or very strong (0.70-1.00). We utilized the chi-square test to assess gender distribution among subgroups. Values are presented as mean and standard deviation (SD). All of the analyses were carried out using SPSS software version 26.0 (SPSS Inc., Chicago, Illinois, USA). A P-value of <0.05 was considered statistically significant. |
|  |  | (*b*) Describe any methods used to examine subgroups and interactions | 9 | We utilized the chi-square test to assess gender distribution among subgroups. We conducted an analysis of variance (ANOVA) to compare cartilage thickness among different subgroups and controls. Following the ANOVA, we utilized the Bonferroni test as the post-hoc test to evaluate specific group differences. |
|  |  | (*c*) Explain how missing data were addressed | - | There were no missing data. |
|  |  | (*d*) *Cohort study*—If applicable, explain how loss to follow-up was addressed  *Case-control study*—If applicable, explain how matching of cases and controls was addressed  *Cross-sectional study*—If applicable, describe analytical methods taking account of sampling strategy | 7 | We utilized G*Power software to calculate the sample size. By inputting the values of alpha and beta error, two tailed significance level, and effect size into the independent t-test formula, the software determined the required sample size for our study. |
|  |  | (*e*) Describe any sensitivity analyses | 10 | We did not conduct sensitivity analyses as our study compared athletes with healthy controls and further analyzed subgroups within these groups. Since there were no variations in assumptions or criteria during the statistical analysis, sensitivity analyses were not necessary. |
| Results | | | | |
| Participants | 13* | (a) Report numbers of individuals at each stage of study—eg numbers potentially eligible, examined for eligibility, confirmed eligible, included in the study, completing follow-up, and analysed | 10 | Numbers potentially eligible: Not specified, examined for eligibility: Not specified, confirmed eligible: 72 athletes (36 in subgroup 1 and 36 in subgroup 2), and 72 controls, included in the study: 72 athletes (36 in subgroup 1 and 36 in subgroup 2), and 72 controls, completing follow-up: Not specified, Analyzed: 72 athletes (36 in subgroup 1 and 36 in subgroup 2) and 72 controls |
|  |  | (b) Give reasons for non-participation at each stage | - | No participants were excluded at any stage of the study. |
|  |  | (c) Consider use of a flow diagram | - | We determined the sample size using a specific formula for cross-sectional studies. As our study did not involve participant exclusions or follow-up, a flow diagram was not utilized. |
| Descriptive data | 14* | (a) Give characteristics of study participants (eg demographic, clinical, social) and information on exposures and potential confounders | 10 | A total of 72 professional athletes with a mean age of 29.6 ± 6.6 and mean BMI of 25.9 ± 3.1 (50 men, 22 women) and 72 healthy controls with a mean age of 31.9 ± 6.7 and mean BMI of 25.6 ± 3.2 (50 men, 22 women) were enrolled in this study. The detailed demographic characteristics of the athletes and controls are shown in Table 1. |
|  |  | (b) Indicate number of participants with missing data for each variable of interest | - | No participants had missing data for any variable of interest. |
|  |  | (c) *Cohort study*—Summarise follow-up time (eg, average and total amount) |  |  |
| Outcome data | 15* | *Cohort study*—Report numbers of outcome events or summary measures over time |  |  |
|  |  | *Case-control study—*Report numbers in each exposure category, or summary measures of exposure |  |  |
|  |  | *Cross-sectional study—*Report numbers of outcome events or summary measures | 10 | Although the athletes had thinner femoral cartilage thickness values at all measured sites compared to the controls, the difference only reached statistical significance in the right lateral (P-value = 0.002), left lateral (P-value = 0.004), and left medial condyles (P-value = 0.039). |
| Main results | 16 | (*a*) Give unadjusted estimates and, if applicable, confounder-adjusted estimates and their precision (eg, 95% confidence interval). Make clear which confounders were adjusted for and why they were included | 11 | When comparing the subgroups of athletes with the controls, the analysis showed significantly thinner cartilage thickness in the right lateral condyle (2.13 ± 0.34 vs. 2.39 ± 0.31, P-value = 0.001), left lateral condyle (2.22 ± 0.31 vs. 2.46 ± 0.35, P-value = 0.005), and left medial condyle (2.21 ± 0.46 vs. 2.42 ± 0.36, P-value = 0.027) in subgroup 2 (≥12 months of treadmill running) compared with the control (Figure 1). No significant difference was observed in cartilage thickness between subgroups 1 and 2 and between subgroup 1 and the control group. |
|  |  | (*b*) Report category boundaries when continuous variables were categorized | 11 | In our study, we categorized participants based on total months of activity into three groups: Control (no physical activity), Subgroup 1 (3 to under 12 months of activity), and Subgroup 2 (12 months or more of activity). The category boundaries correspond to the thresholds for each subgroup, with participants falling into the appropriate category based on their total months of activity. |
|  |  | (*c*) If relevant, consider translating estimates of relative risk into absolute risk for a meaningful time period | - | In our study, absolute risk and relative risk were not calculated. |

Continued on next page

| Other analyses | 17 | Report other analyses done—eg analyses of subgroups and interactions, and sensitivity analyses | 11, 12 | **Normality Test**: The normality of our data was assessed by conducting the Shapiro-Wilk test. **Demographic Characteristics**: We found no significant differences between the groups regarding age, sex, weight, height, BMI, and smoking status (P > 0.05). **Correlation Analysis**: We observed a significant negative correlation between the duration of treadmill running and distal femoral cartilage thickness in the right lateral (r = -0.236, P-value = 0.046) and left lateral condyles (r = -0.233, P-value = 0.049), suggesting that as the duration of treadmill running increases, the thickness of cartilage decreases. However, no significant correlation was found between distal femoral cartilage thickness at different sites and the athletes' age, body mass index, speed and incline of treadmill running, and minutes of activity per session and week (P-value > 0.05). **Group Differences**: Female athletes had significantly thinner distal femoral cartilages in all measured areas than male athletes (P-value < 0.05). |
| --- | --- | --- | --- | --- |
| Discussion | | | | |
| Key results | 18 | Summarise key results with reference to study objectives | 12 | Our findings revealed a negative correlation between the total months of treadmill running and distal femoral cartilage thickness in different knee regions among physique bodybuilders. Notably, athletes with over one year (12 months) of treadmill running experience exhibited significantly thinner cartilage thickness in most knee regions compared to non-athlete controls. No significant correlations were observed between cartilage thickness and treadmill incline, speed, participant BMI, or mean duration of activity per session. |
| Limitations | 19 | Discuss limitations of the study, taking into account sources of potential bias or imprecision. Discuss both direction and magnitude of any potential bias | 15 | Relying solely on a single methodological approach may limit the depth of our analysis. Incorporating multi-modal research methods could offer a more comprehensive understanding of the phenomenon under investigation. For example, histological and molecular evaluations using multiple imaging modalities may improve understanding of the topic and help to explore the underlying mechanisms sufficiently. However, we must consider such assessments' ethical issues, costs, and challenges when dealing with human participants. In addition, despite our efforts to apply strict inclusion and exclusion criteria, controlling all variables is challenging. For example, treadmill devices may have different shock absorbent capabilities. It's also crucial to highlight the potential impact of other possible limited lower extremity physical activity and the indirect effects of upper extremity activity on knee cartilage through biomechanical and systemic responses. However, such impacts seem minimal and inconclusive. Most importantly, the present study was retrospective. Although we attempted to utilize documented information with the assistance of their coaches while collecting information regarding athletes’ physical activity, it is important to acknowledge that the possibility of recall bias cannot be completely excluded. so future longitudinal studies with prolonged follow-ups are necessary to fix the limitations and draw cause-and-effect conclusions between vigorous-intensity treadmill running and cartilage thickness changes. |
| Interpretation | 20 | Give a cautious overall interpretation of results considering objectives, limitations, multiplicity of analyses, results from similar studies, and other relevant evidence | 12, 15 | The findings suggest a negative correlation between the duration of treadmill running and distal femoral cartilage thickness among physique bodybuilders. Notably, athletes with over one year of treadmill running experience exhibited significantly thinner cartilage compared to non-athlete controls. These results underscore the potential impact of prolonged treadmill running on knee cartilage health. However, caution is warranted due to limitations such as reliance on a single method, possible biases in retrospective data collection, and the challenge of controlling all variables. Further research, including longitudinal studies and diverse methodologies, is needed to confirm these findings and elucidate the underlying mechanisms. |
| Generalisability | 21 | Discuss the generalisability (external validity) of the study results | 12-15 | This study focused on physique athletes who engage in vigorous treadmill running, selected from sport clubs. The evaluation of knee cartilage thickness was conducted using ultrasonography, a method previously employed among other populations to measure cartilage thickness. However, it's essential to recognize that the study's population and methodology may limit the generalizability of the findings to broader populations or contexts. Similar studies assessing cartilage health among individuals engaging in vigorous physical activity have yielded varied results, with some reporting detrimental effects and others finding no significant impact. Therefore, caution is warranted when extrapolating these findings to different populations or settings.  Future longitudinal studies may be necessary to address potential biases inherent in this study design and provide more robust evidence regarding the impact of vigorous treadmill running on knee cartilage thickness across diverse populations and over extended periods of time. |
| Other information | |  | | |
| Funding | 22 | Give the source of funding and the role of the funders for the present study and, if applicable, for the original study on which the present article is based | 17 | No funding was received. |

*Give information separately for cases and controls in case-control studies and, if applicable, for exposed and unexposed groups in cohort and cross-sectional studies.

**Note:** An Explanation and Elaboration article discusses each checklist item and gives methodological background and published examples of transparent reporting. The STROBE checklist is best used in conjunction with this article (freely available on the Web sites of PLoS Medicine at http://www.plosmedicine.org/, Annals of Internal Medicine at http://www.annals.org/, and Epidemiology at http://www.epidem.com/). Information on the STROBE Initiative is available at www.strobe-statement.org.
